# Epidemiology of eye diseases: outcomes from a free provincial eye clinic in Papua New Guinea

**DOI:** 10.3389/fmed.2023.1272337

**Published:** 2023-12-18

**Authors:** Bismark Owusu-Afriyie, Theresa Gende, Frederick Silki, Bolgii Ishmael, Joelda Kuiaha

**Affiliations:** ^1^Faculty of Medicine and Health Sciences, Divine Word University, Madang, Papua New Guinea; ^2^The Fred Hollows Foundation NZ, Auckland, New Zealand; ^3^The Fred Hollows Foundation PNG Inc., Madang, Papua New Guinea

**Keywords:** cataract, cornea, refractive error, pattern, eye disease, epidemiology, ocular surface

## Abstract

**Aim:**

To ascertain the prevalence and pattern of eye problems in Madang Province, Papua New Guinea.

**Materials and methods:**

A six-month retrospective study was performed at Madang Provincial Hospital Eye Clinic. Convenience sampling was used in this study and all patient records from January to June 2020 were included. Data was extracted using Microsoft Excel and the data included gender, age, occupation, district where the patient lived, presenting visual acuity, and diagnosis. It was then analyzed using International Business Machines Corporation’s Statistical Package for the Social Sciences version 26. A *p*-value of ≤0.05 was considered statistically significant.

**Results:**

A total of 1,715 patients received services at the eye clinic between January and June 2020, and 1,664 were included in this study. The mean age of the patients was 39.3 ± 20.3 years. There were slightly more males (50.4%) than females. The overall leading ocular morbidities were corneal ulcers and keratitis (20.7%), refractive errors (17.4%), and cataracts (16.8%). More than half of the patients (56.2%) were either visually impaired or blind. Nearly half of the patients (41.8%) traveled long distances to seek services at the eye clinic. There was a significant association between demographic characteristics, diagnosis, and level of visual impairment.

**Conclusion:**

There is a high prevalence of potential causes of visual impairment and blindness in Madang Province and these conditions affect all age groups and genders. It is essential to increase accessibility to eye care services in the country.

## 1 Introduction

The causes of ocular and visual problems differ across communities and populations (1). Factors such as age, gender, occupation, ethnicity, and prevailing medical conditions have been reported to significantly contribute to the risks, prevalence, and distribution of various ocular morbidities (1–5). For instance, studies have reported a high prevalence of conjunctival disorders (4, 6–9) and corneal abnormalities (7, 8, 10) among welders and farmers, and being a female aged ≥35 years was significantly associated with ocular disorders among welders (4). Monsudi et al. (11) also reported high levels of cataracts, refractive errors, and glaucoma in a rural community in Nigeria during a free eye outreach.

The requirements for eye care services vary according to the diagnosis and severity of the condition. Nonetheless, the effective management of ophthalmic patients depends on early diagnosis and treatment of morbidities to prevent blindness and vision loss. Studies have been conducted in various populations across the globe to understand the pattern and trends of eye diseases and suggest measures for new and improved services (2, 8, 9, 11–13).

In Papua New Guinea (PNG), a rapid assessment of avoidable blindness (RAAB) was carried out in 2017 but the study focused mainly on the causes of visual impairment (VI) and blindness (presenting visual acuity <6/12) in the older population (aged 50 years and older) (14). Contrariwise, studies have indicated that there are potentially blinding conditions that do not present significant ocular or visual symptoms in the initial stages (11, 15), and PNG is not immune to such potential causes of visual impairment (16, 17). Therefore, the findings of the RAAB are limited in terms of the general presentation of eye disorders in the country. In addition, the RAAB did not address the causes of VI and blindness in children and the working-age population, but this age group constitutes a high proportion of the population of PNG (18).

The absence of data on the prevalence and distribution of ocular morbidities across all age groups in PNG is worrisome. This study was conducted using six-month patients’ records at Madang Provincial Hospital Eye Clinic to understand the prevalence and trends of eye problems across all age groups in the province. The findings of this study would provide evidence to streamline eye care services in the direction of preventive and curative eye care programs in PNG and similar countries.

## 2 Materials and methods

### 2.1 Study setting

The study was carried out at Madang Provincial Hospital Eye Clinic. It was the only eye care centre in the province at the time of this study, and it provides free eye examinations, pharmacological treatments, and surgical services. These services are funded by the Fred Hollows Foundation New Zealand. The clinical services at the facility are provided by an ophthalmologist, ophthalmic clinicians, supporting nurses and community health workers. The eye clinic also serves as a referral centre for nearby provinces and helps in training medical, ophthalmic, nursing and health extension students in the country.

### 2.2 Study design and data collection procedure

This was a hospital-based retrospective study. Convenience sampling was used in this study. This sampling method was used because the study involved all patient records at the eye clinic for the period under study. Data collection involved the use of Microsoft Excel to extract and record socio-demographic and clinical data. The specific data extracted included gender, age, occupation, district where the patient lived, ocular and medical history, presenting visual acuity and diagnosis.

### 2.3 Inclusion and exclusion criteria

All patient records at the eye clinic were reviewed and only those who were examined and diagnosed from January to June 2020 were included in the study. The study excluded patients whose diagnosis and records were inconclusive and those whose eye examination revealed no ocular or visual defect.

### 2.4 Consent to participate and publish

The study involved the use of secondary data. Patients who visit the facility are made aware that data could be used for research purposes since it is also a training centre for eye care professionals and medical students. The management of the eye clinic granted permission in writing before the data was collected. No identifiable information was collected from the records.

### 2.5 Study definitions

The operational definitions adopted for VI in this study were based on the patients’ presenting visual acuity (VA) in the better eye (19).

No VI: VA ≥ 6/12 in the better eye
Mild VI: VA <6/12 to ≥6/18 in the better eye
Moderate VI: VA <6/18 to ≥6/60 in the better eye
Severe VI: VA <6/60 to ≥3/60 in the better eye
Blindness: VA < 3/60 in the better eye

### 2.6 Data analysis

The extracted data was analyzed using International Business Machines Corporation’s Statistical Package for the Social Sciences (IBM SPSS) version 26 (IBM Corp., Armonk, N.Y., USA). Data were summarized using appropriate descriptive statistics such as frequencies and percentages for categorical variables while continuous variables were presented as means and standard deviations. Associations were drawn using Pearson’s chi-square test for independence and distributions. All data were analyzed at 95% confidence interval. A *p*-value of ≤0.05 was considered statistically significant.

## 3 Results

A total of 1,715 patients visited the eye clinic between January and June 2020 out of which 1,664 met the inclusion criteria for this study. The excluded records were those of 33 patients whose ocular and visual examination revealed no abnormality and 18 patients whose records were incomplete.

### 3.1 Demographic characteristics of patients

#### 3.1.1 Age, gender, and occupation

The mean age of the patients was 39.3 ± 20.3 years and ranged from 2.0 weeks to 86.0 years. Most of the patients (18.4%) were ≥ 61 years. There were slightly more males (50.4%) than females. The age and gender distributions are as detailed in Table 1. The occupation of most of the patients (88.6%) was not recorded; therefore, no associations were analyzed between occupation and other variables. Students (5.2%) were the highest recorded occupation. The other recorded occupations were lecturers, sales officers, welders, carpenters, mechanics, and receptionists (2.8%).

**Table 1:** Age and gender distribution of patients (*n* = 1,664).

		Gender of patients		Total (%)
		Male	Female	
Age group (years)	≤ 10	72	77	149 (9.0)
	11–20	99	114	213 (12.8)
	21–30	122	148	270 (16.2)
	31–40	111	123	234 (14.1)
	41–50	124	140	264 (15.9)
	51–60	116	111	227 (13.6)
	≥ 61	195	112	307 (18.4)
Total		839 (50.4)	825 (49.6)	1664 (100)

#### 3.1.2 Residential provinces and districts

The majority of the patients (92.6%) were from Madang Province followed by Morobe Province (3.1%) which is the nearest province to Madang Province by distance. There were only a few patients who reported from the other 12 provinces (less than 1.0% from each of these provinces: Western Highlands, East Sepik, New Ireland, Eastern Highlands, Chimbu, Sandaun, Manus, Southern Highlands, Hela, National Capital District, East New Britain, and West New Britain). In addition, more than half of all the patients were residents of Madang District. The distribution of the provinces and districts are shown in Table 2.

**Table 2:** Distribution of patients by their provinces and districts (*n* = 1,664).

Province	District	Frequency (%)
Madang province	Madang	969 (58.2)
	Sumkar	244 (14.7)
	Middle Ramu	110 (6.6)
	Raicoast	83 (5.0)
	Bogia	81 (4.9)
	Usino Bundi	54 (3.2)
Morobe province	Lae	40 (2.4)
	Markham	4 (0.2)
	Finchaffen	2 (0.1)
	Menyamya	1 (0.1)
	Wau Bulolo	3 (0.2)
Eastern Highlands	Goroka	5 (0.3)
	Kainantu	4 (0.2)
Simbu province	Chimbu	2 (0.1)
	Kundiawa	2 (0.1)
East Sepik	Wewak	17 (1.0)
	Angoram	5 (0.3)
	Maprik	1 (0.1)
Manus province	Manus	1 (0.1)
Western Highlands province	Mt. Hagen	10 (0.6)
National Capital District	Port Moresby	7 (0.4)
New Ireland Province	Kavieng	2 (0.1)
Southern Highlands	Tari	2 (0.1)
	Mendi	1 (0.1)
East New Britain	Kokopo	1 (0.1)
West New Britain	Kimbe	1 (0.1)
West Sepik (Sandaun)	Aitape	3 (0.2)
	Vanimo	1 (0.1)
Enga	Wabag	9 (0.5)
Hela	Tari	2 (0.1)
Total		1664 (100)

### 3.2 Self-reported medical and ocular history

One thousand five hundred and eighty-eight (95.4%) patients reported that they did not have any previous eye or vision problem. Similarly, 1,557 (93.6%) patients reported that they had never been diagnosed of any medical condition. Therefore, no associations were analyzed between their health history and any other variable. The most reported ocular history was refractive errors (3.8%), and tuberculosis (1.7%) was the highest reported medical condition.

### 3.3 Clinical data and associations

#### 3.3.1 Category of visual impairment (VI)

The level of VI of 52 (3.1%) patients could not be categorized because their visual acuities were not recorded. There was an equal number of males and females whose VI could not be categorized. The distribution of VI among the patients are shown in Table 3. More than one-third of the patients had no impairment of vision and about a quarter (23.1%) of the patients had severe VI. More females (23.3%) had no impairment of vision than males (16.3%). On the other hand, there were higher proportions of male patients with visual impairment and blindness than female patients. Older patients (≥ 61 years) had disproportionately higher rates of severe VI while larger proportions of patients below 61 years of age had no impairment of vision.

**Table 3:** Pattern of visual impairment according to gender, age, and type of oculo-visual condition (*n* = 1,664).

Demographics		Category of visual impairment						Total	*p*-value
		No visual impairment (≥ 6/12)	Mild Visual Impairment (< 6/12 ≥ 6/18)	Moderate Visual Impairment (< 6/18 ≥ 6/60)	Severe Visual Impairment (< 6/60 ≥ 3/60)	Blindness (< 3/60)	Cannot be categorized		
Gender	Male	272	160	100	246	36	25	839(50.4)	<0.001
	Female	405	141	95	138	19	27	825 (49.6)	
	Total (%)	677 (40.7)	301 (18.1)	195 (11.7)	384 (23.1)	55 (3.3)	52 (3.1)	1664 (100)	
Age group (years)	≤ 10	32	10	11	26	22	48	149(8.9)	<0.001
	11–20	110	34	15	46	7	1	213(12.8)	
	21–30	171	45	26	25	3	0	270(16.2)	
	31–40	137	46	20	26	3	2	234(14.1)	
	41–50	124	60	25	53	2	0	264(15.9)	
	51–60	66	53	39	63	5	1	227(13.6)	
	≥ 61	37	53	59	145	13	0	307(18.4)	
	Total (%)	677 (40.7)	301 (18.1)	195 (11.7)	384 (23.1)	55 (3.3)	52 (3.1)	1664 (100)	
Type of oculo-visual condition	Conjunctival abnormalities	208 (12.5)	43	14	16	7	14	302(18.1)	<0.001
	Corneal abnormalities	191 (11.5)	104 (6.25)	41	59	5	16	416(25.0)	
	Uvea abnormalities	8	13	16	14	2	0	53(3.2)	
	Lens abnormalities	9	19	57	199 (12.0%)	12	0	296(17.8)	
	Refractive errors	142 (8.5)	85	40	12	1	1	281(16.9)	
	Retinal abnormalities	4	5	12	17(1.0)	4	5	47(2.8)	
	Glaucoma and optic neuropathies	3	0	3	16 (1.0)	4	0	26(1.6)	
	Eyelid abnormalities	74 (4.4)	17	3	5	3	8	110(6.6)	
	Vitreous abnormalities	4	0	0	7	3	0	14(0.8)	
	Scleral and episcleral abnormalities	19	3	1	0	0	0	23(1.4)	
	Other abnormalities	15	12	8	39	14	8	96(5.8)	
	Total (%)	677 (40.7)	301 (18.1)	195 (11.7)	384 (23.1)	55 (3.3)	52 (3.1)	1664(100)	

#### 3.3.2 Diagnosis

Corneal ulcers and keratitis (20.7%) were the main eye problems diagnosed among the patients. This was closely followed by refractive errors (17.4%) and cataracts (16.8%). The main ocular problems among the 149 children aged ≤10 years were conjunctivitis (22.8% of them), corneal ulcers and keratitis (19.5% of them), and chemical injury (12.8% of them). Cataract (50.4%) and refractive error (17.6%) were the most common conditions among the older patients (≥ 61 years) in this study.

Due to the large number of different diagnoses among the patients, we further grouped the patients into younger patients (≤ 20 years) and adult patients (≥ 21 years) to determine the relationship of diagnosis with their age and gender (Table 4). Male patients had significantly higher proportion of cataract and blunt trauma compared to female patients (*p* < 0.001 and 0.045, respectively). On the other hand, female patients were more likely to present with pterygium and conjunctivitis than male patients (*p* = 0.002 and 0.003, respectively). The adult patients recorded significantly higher rates of refractive errors, cataract, corneal opacities and scarring, uveitis, corneal ulcers and keratitis, pseudophakia, pterygium and dry eyes (all *p* < 0.05) compared to the younger patients.

**Table 4:** Distribution of diagnosis according to gender and age (*n* = 1664).

Diagnosis	Gender				Age group			
	Male	Female	Total (%)	*p*-value	≤ 20 years	≥ 21 years	Total (%)	*p*-value
Refractive errors	129	161	290 (17.4)	0.094	24	266	290(17.4)	< 0.001
Cataract	182	97	279 (16.8)	<0.001	7	272	279(16.8)	< 0.001
Corneal opacities and scarring	20	17	37(2.2)	0.628	12	25	37(2.2)	0.039
Blunt trauma	46	28	74 (4.4)	0.045	31	43	74 (4.4)	0.174
Uveitis	37	23	60(3.6)	0.070	21	39	60(3.6)	0.026
Pseudophakia	17	25	42(2.5)	0.041	4	38	42(2.5)	< 0.001
Corneal ulcers and keratitis	163	181	344(20.7)	0.266	80	264	344(20.7)	< 0.001
Pterygium	41	75	116(7.0)	0.002	4	112	116(7.0)	< 0.001
Conjunctivitis	53	90	143(8.6)	0.003	79	64	143(8.6)	0.234
Eyelid laceration	6	5	11(0.7)	0.7412	6	5	11(0.7)	0.741
Dry Eyes	9	20	29(1.7)	0.056	4	25	29(1.7)	0.002
Blepharitis	5	10	15(0.9)	0.212	8	7	15(0.9)	> 0.050
Glaucoma and optic neuropathies	17	9	26(1.6)	0.144	9	17	26(1.6)	0.144
Chemical injury	17	15	32(1.9)	0.735	21	11	32(1.9)	0.084
Corneal laceration	9	10	19(1.1)	0.794	14	5	19(1.1)	0.058
Scleritis and episcleritis	12	15	27(1.6)	0.535	9	18	27(1.6)	0.094
Chalazion	6	6	12(0.7)	1.000	7	5	12(0.7)	> 0.050
Conjunctival granuloma	4	7	11(0.7)	0.370	2	9	11(0.7)	*
Diabetic retinopathy	10	12	22(1.3)	0.640	1	21	22(1.3)	*
Maculopathies	7	3	10(0.6)	0.240	0	10	10(0.6)	*
Subconjunctival hemorrhage	4	3	7(0.4)	*	2	5	7(0.4)	*
Dacryocystitis	0	1	1(0.06)	*	0	1	1(0.06)	*
Retinoblastoma	5	1	6(0.4)	*	6	0	6(0.4)	*
Conjuctival foreign body	4	2	6(0.4)	*	0	6	6(0.4)	*
Corneal foreign body	7	1	8(0.5)	*	2	6	8(0.5)	*
Subluxated lens	2	1	3(0.2)	*	0	3	3(0.2)	*
Entropion	0	2	2(0.1)	*	0	2	2(0.1)	*
Retinal vein occlusion	1	0	1(0.06)	*	0	1	1(0.06)	*
Ptosis	3	0	3(0.2)	*	0	3	3(0.2)	*
Microphthalmia	2	1	3(0.2)	*	3	0	3(0.2)	*
Retinal detachment	6	0	6(0.4)	*	0	6	6(0.4)	*
Hypertensive retinopathy	2	1	3(0.2)	*	0	3	3(0.2)	*
Vitreous haemorrhage	4	0	4(0.2)	*	0	4	4(0.2)	*
Nasolacrimal duct obstruction	2	1	3(0.2)	*	2	1	3(0.2)	*
Endophthalmitis	1	1	2(0.1)	*	0	2	2(0.1)	*
Preseptal cellulitis	2	0	2(0.1)	*	1	1	2(0.1)	*
Bell’s palsy	1	0	1(0.06)	*	0	1	1(0.06)	*
Posterior vitreous detachment	2	0	2(0.1)	*	2	0	2(0.1)	*
Tropia	1	0	1(0.06)	*	1	0	1(0.06)	*
Squamous cell carcinoma	0	1	1(0.06)	*	1	0	1(0.06)	*
Total (%)	839 (50.4)	825(49.6)	1,664(100.0)		362(21.8)	1,302(78.2)	1,664(100.0)	

^*^Denotes fewer frequencies where *p*-values were not computed.

## 4 Discussion

We found that ocular surface problems, refractive errors, cataract, blunt trauma, and chemical injuries were the leading diagnoses among the patients. This study reported that slightly more males than females visited the eye clinic. This contrasts the findings from a free eye clinic in an urban state hospital in Nigeria (20), and also during a free outreach in a rural community in Nigeria (11). Nonetheless, Ajaiyeoba and Scott (1) reported male dominance in their study. The difference could be attributed to the different settings in which the studies were conducted. Moreover, males are more likely to be gainfully employed in many societies (1), and hence they can afford associated costs such as transport and feeding when they are seeking eye care services. This may have contributed to the relatively higher number of male patients in our study.

In spite of the male preponderance in the hospital attendance, there were more females (23.3%) with no impairment of vision than males (16.3%). In this study, cataract, glaucoma, retinal detachment, corneal opacities, maculopathy, chemical injury, trauma, and uveitis were more commonly reported among male patients (see Table 4). These conditions are potential causes of VI and permanent loss of sight (10, 21–24). Our findings rationalize with that of another study that indicated that ocular trauma is more common among males (25). D’Oria et al. (26) also recently reported a high ratio of males to females in an ocular emergency study that used reduced vision, eye trauma, and the presence of floaters and flashes as some of the predictors of urgency at an ophthalmic emergency room. Thus, the presence of significant visual symptoms may have also influenced the high uptake of eye care services by the males in the current study. A further study is needed to explain the high rates of these eye diseases among the male population, likewise the significantly large numbers of pterygium and conjunctivitis among females in Madang and PNG.

In the current study, the majority of the patients (78.2%) were adults (≥ 21 years). Notably, there is evidence suggesting low uptake of eye care services by children and the reason for this is that children are unable to express themselves enough especially when the symptoms are mild (1, 20). This leads to delayed intervention until they mature or when the symptoms are more severe. The present finding yielded important insight into this situation. There is less focus on pediatric eye care services in PNG. Currently, there is no eye examination required for admission into schools, and school vision screening programs are often neglected in many parts of the country. Parents and guardians mostly send their child (ren) for eye examination after the child’s vision is compromised or when there is a severe eye infection. These factors account for the low uptake of eye care services by the younger population. Therefore, we propose that awareness programs should be intensified among parents, teachers, and guardians about the need for children to seek regular eye check-ups to preserve their sight and reduce under diagnosis of eye diseases among children. This current study subsequently identified that patients aged ≥41 years had higher rates of severe VI. This is similar to other studies as increasing age has been associated with severity of VI (12, 27–31).

The results on VI in this study compares to the global trend on the causes of VI and blindness (32). A larger portion of the patients who were diagnosed with refractive errors, conjunctival and eyelid abnormalities had no impairment of vision (8.5, 12.5 and 4.4% respectively). On the other hand, most of the patients diagnosed of glaucoma, retinal and lens disorders had severe VI (1.0, 1.0 and 12.0% respectively). The findings agree with that of Maake and Oduntan (31) who reported glaucoma and cataract as the main causes of VI and blindness in Nkhensani Hospital Eye Clinic in South Africa. Similarly, Alswailmi (33) reported that cataract, uncorrected refractive errors and glaucoma were the most common causes of VI in Saudi Arabia. In addition, the outcome of this current study supports the RAAB conducted in PNG in which refractive error was the leading cause of early VI, and cataract was the most significant cause of severe VI and blindness (14). The prevalence of retinal disorders in the present study was very low (1.0%) compared to studies in Nepal (52.4%) and Malaysia (11.5%) (12, 34). The difference may be attributed to the different study designs, criteria for diagnosis and the age of participants in the other two studies.

The leading eye problems among the younger patients (≤ 20 years) were corneal ulcers and keratitis, and conjunctivitis (Table 4). Hospital based studies have similarly reported conjunctivitis and eye infections as the main ocular morbidities among children (2, 20, 25, 35). These preventable eye diseases can lead to absenteeism in school (35) and poor academic performance among affected children. Demissie and Demissie (2) have suggested that vision is essential in the early years of life for learning and communication, therefore children should be educated and guided on proper hygiene, and to abstain from activities that could harm their eyes thereby contributing toward the onset and progression of eye problems.

The adult patients in this study recorded large numbers of corneal ulcers and keratitis and refractive error as the main ocular problems (Table 4). In Madang and PNG, most of the people conduct their daily activities in an open environment engaging in farming (16, 36), trading (36), welding and carpentry without using protective eyewear. In addition, tribal conflicts and domestic violence are common. Thus, they have direct exposure to vegetation and conditions that put them at risk of corneal problems. A large proportion of affected individuals assume that eye diseases are self-limiting and/or could be treated at home. Therefore, their first line of action is not a hospital visit. Nearly all patients who recently visited the Madang Provincial Hospital Eye Clinic reported that they only sought for eye care services when their vision was threatened (36). Corneal ulcers and keratitis are potential sight threatening morbidities (10, 37, 38), therefore it is necessary that the public are well educated about the benefits of protective eyewear and timely reporting of eye problems to prevent blindness associated with cornea disorders as this may lead to significant loss of productivity in the country.

The prevalence of refractive error among the older patients (≥ 61 years) in this study compares to that of a similar hospital-based study in North-East India (39). Nonetheless, the prevalence of cataract and refractive error among these patients in this study was higher than that of the study by Reddy et al. (40) who recorded lower prevalence of refractive error (10.8%) and cataract (32.9%) at University of Malaya Medical Centre. Studies have reported increasing age as a contributing factor for cataract (3, 27, 41), therefore it is not unexpected that there is high rate of cataract in the older patients in the current study. The prevalence of cataract in this study is however lower than that of a hospital-based study in Ethiopia (57.0%) (42). Contrariwise, other developing countries have recorded much lower prevalence of cataract in the older populations (3, 43, 44). The differences may be attributed to variations in study designs and variations in the exposure to environmental conditions at the different geographical settings. In general, there was a disparity between the number of patients who had a history of refractive error (3.8%) and those diagnosed with refractive error (17.4%) in the current study. Refractive errors can be corrected by a simple pair of spectacles; however, cost of seeking services and poor access to available services may be the barriers to the uptake of refractive error interventions in PNG.

The eye clinic is strategically located within the provincial hospital in Madang; therefore, it is not surprising that most of the patients were residents of Madang District (58.2%) since the clinic is readily accessible to them. We were intrigued by the number of patients who traveled long distances from other districts (41.8%) and provinces (7.4%) to seek services at the eye clinic. This is because there are only a few eye clinics in the country. Having provided information about the epidemiology of ocular and visual morbidities in PNG, we advocate for measures to increase accessibility to eye care services across the country.

There was no detailed information about the grade or stage of the diseases recorded in the patients’ folders (for example, diagnosis was recorded as diabetic retinopathy instead of proliferative or non-proliferative diabetic retinopathy). Nonetheless, this does not substantially affect the measures we have recommended for improved accessibility and delivery of eye care services in PNG. In addition, specific microbial isolates were not recorded for conditions such as keratitis. We suggest that clinicians should routinely investigate the etiology of ocular infections in a quest to deliver patient-centered eye care services, and to reduce the risk of microbial resistance to antibiotics (45). The study was delimited to ophthalmic patients who were seeking eye care services at the eye clinic. Notwithstanding, we do not anticipate that the findings at community level (especially in Madang District and Madang Province) will differ significantly from our report due to the large number of patients’ records that were reviewed in this study.

## 5 Conclusion

To summarize, this study revealed that demographic characteristics such as age and gender have significant influence on ocular morbidities and VI in Papua New Guinea. Corneal problems, refractive errors and cataract are the most common eye and vision problems in Madang and these conditions affect all age groups and genders. Given the high prevalence of ocular surface conditions in the population, it is essential to strengthen corneal services in the country. A community-based study across all age groups in the country is highly recommended to better understand the trends of ocular and visual disorders in PNG and explain the factors that influence the gender variations in the prevalence of eye problems in the country.

## Data availability statement

The data analyzed in this study is subject to the following licenses/restrictions: Data for this current study will be made available upon reasonable request form the authors. Requests to access these datasets should be directed to BO-A, dr.bismarkoa@gmail.com.

## Ethics statement

The studies involving humans were approved by the Faculty of Medicine and Health Sciences Research Committee of Divine Word University, Madang. The studies were conducted in accordance with the local legislation and institutional requirements. Written informed consent for participation was not required from the participants or the participants’ legal guardians/next of kin in accordance with the national legislation and institutional requirements.

## Author contributions

BO-A: Conceptualization, Data curation, Formal analysis, Methodology, Writing – original draft, Writing – review & editing. TG: Data curation, Methodology, Resources, Writing – review & editing. FS: Data curation, Formal analysis, Investigation, Writing – review & editing. BI: Data curation, Formal analysis, Investigation, Writing – review & editing. JK: Data curation, Formal analysis, Investigation, Writing – review & editing.
